# Socio-behavioral risk factors among older adults living with HIV in Thailand

**DOI:** 10.1371/journal.pone.0188088

**Published:** 2017-11-14

**Authors:** Patou Masika Musumari, Arunrat Tangmunkongvorakul, Kriengkrai Srithanavibooncha, Mitchell D. Feldman, Wathee Sitthi, Kittipan Rerkasem, Teeranee Techasrivichien, S. Pilar Suguimoto, Masako Ono-Kihara, Masahiro Kihara

**Affiliations:** 1 Department of Global Health and Socio-epidemiology, Kyoto University School of Public Health, Kyoto, Japan; 2 Research Institute for Health Sciences, Chiang Mai University, Chiang Mai, Thailand; 3 Faculty of Medicine, Chiang Mai University, Chiang Mai, Thailand; 4 Division of General Internal Medicine, University of California San Francisco, San Francisco, California, United States of America; 5 Center for Medical Education, Graduate School of Medicine, Kyoto University, Kyoto, Japan; Thai Red Cross AIDS Research Centre, THAILAND

## Abstract

**Background:**

There has been a global increase in HIV infection in persons 50 years of age and older. This group is at risk for development of chronic illness that may be exacerbated by socio-behavioral risk factors such as smoking, unhealthy alcohol use, and sedentary lifestyle. However, socio-behavioral risk factors in this older HIV infected population are not well described. The current study aims to describe and document factors related to alcohol use, tobacco smoking, and physical exercise in older adults living with HIV (OALHIV).

**Methods:**

This cross-sectional quantitative study was conducted between August and September 2015, and enrolled HIV-infected participants aged 50 years and older from 12 community hospitals in Chiang Mai Province, Northern Thailand.

**Results:**

Of the 364 participants recruited in the study, 57.1% were female, and 67.3% were between 50–59 years of age. Respectively, 15.1%, 59.1%, and 18.7% were current smokers, currently engaged in physical exercises, and reported ever drank alcohol in the past year. 22.1% of those who drank alcohol reported experience of heavy episodic drinking. Male gender was one of the strongest predictors of ever drank alcohol in the past year (AOR, 4.66; CI, 2.28–9.49; *P*<0.001) and of being a current smoker (AOR, 13.41; CI, 7.23–24.87; *P*<0.001). Lower household income was associated with increased odds of ever drank alcohol in the past year (household income (1 USD = 35 THB) of ≤ 5,000 Baht versus > 20,000 Baht: AOR, 5.34; CI, 1.28–22.25; *P* = 0.021). Lower educational level was associated with decreased odds of physical exercises (no education versus secondary and higher: AOR, 0.22; CI, 0.08–0.55; *P* = 0.001).

**Conclusion:**

Smoking and alcohol use is common among OALHIV, with a substantial proportion not engaging in physical exercises. Interventions for OALHIV should particularly target males and those of lower socio-economic status to deter smoking and alcohol use and to promote physical exercises.

## Introduction

Antiretroviral (ARV) medications are extending the lifespan of HIV-infected persons and this longevity means these older adults with HIV are developing chronic diseases. Health care delivery for older adults with HIV has shifted from acute to chronic care, making it more important to focus on health behavior of the patients [1–3]. Socio-behavioral risk factors, including smoking, alcohol use, and physical inactivity are associated with many chronic non-communicable diseases, and have become significant causes of morbidity and mortality among HIV-infected individuals in areas with large access to antiretroviral medication [4–17].

Older adults living with HIV (OALHIV), defined as HIV-infected individuals aged 50 years and older, account for approximately 10% of adults living with HIV in low- and middle-income countries (LMIC) [18]. However, to date, very little is known about the extent of, and factors associated with alcohol use, smoking and physical activity among OALHIV in LMIC. Most knowledge in this area is derived from literature in high-income countries [18–24].

The prevalence of HIV in individuals aged 50 years and older is not routinely reported in the national statistics in Thailand, the setting of the current study. The literature however suggests that HIV-infected individuals in Thailand will approach older age at a pace faster and at a size larger than other developing countries. This is because of both the severity of the HIV/AIDS epidemic in the past [25–27] and the early initiation of an antiretroviral treatment (ART) program, which covered a large number of HIV-infected people with a high rate of retention rate in care [28, 29].

No study in Thailand has investigated alcohol use and tobacco smoking, and physical activity among OALHIVs. These socio-behavioral risk factors are however reported for the elderly Thai in the general population. In Northern Thailand, 25.2% of adults aged 50 years and older were daily alcohol users and 64% were lifetime alcohol users [30]. Results from the Thailand National Health Examination Survey IV (NHES IV), a nationally representative cross-sectional survey conducted from 2008–2009, showed that the prevalence of Thai population, aged 45 and older, that had consumed alcohol in the past 12 months, were current smokers, and had at least moderate physical activity during leisure time was respectively 29.4%, 19.5%, and 23.7% [31].

In the current study, we describe and provide factors associated with alcohol use, smoking, and physical exercise among HIV-infected older adults in Chiang Mai, Thailand.

## Methods

### Study design, participants, & setting

This study draws on data from our cross-sectional survey on HIV-infected and non-infected older adults. The study was conducted between August and September 2015 in Chiang Mai province, Thailand. Chiang Mai is located in Northern Thailand, and is administratively divided into 25 districts. The province is home to a total of 24 community hospitals, offering general health services and services related to HIV/AIDS prevention, care and treatment. The present report specifically focuses on HIV-infected individuals, aged 50 years or older, receiving ambulatory care in selected facilities. The study was conducted in the 12 district hospitals that serviced the largest population of HIV-infected patients. The number of HIV/AIDS patients in the selected hospitals ranged from 300 to 1,128 at the time prior to the study. The 30 oldest HIV patients registered at each hospital were invited to participate in the study by their care givers (usually a nurse from the ART clinic). The patients who were interested in participating were given an appointment to meet with study staff.

### Data collection instruments and variables

Data were collected through face-to-face interviews, medical records (HIV diagnosis, history of opportunistic infections, plasma viral loads, and information related to ARV medication), and onsite clinical examination (e.g. body mass index (BMI) and waist circumference). The interviews were conducted in Thai using a structured questionnaire which included items on socio-economic and demographic characteristics (gender, age, educational level, occupation, marital status, household monthly income, perceived sufficiency of household monthly income), religion, living conditions, and health behavior information (frequency and intensity of physical exercise, tobacco smoking, and alcohol use).

#### Description of behavioral variables

The behavioral variables were comprised of “alcohol use”, “smoking”, and “physical exercise”.

Alcohol use: The outcome variable “alcohol use” was measured with the item “Have you ever drunk alcohol in the past year?” with “yes and no” as response options. We also used the Alcohol Use Disorders Identification Test (AUDIT) to identify harmful patterns of drinking behavior among the participants who reported having drunk alcohol in the past year. Based on the AUDIT, participants with a history of drinking alcohol were classified into: 1) low risk drinkers, 2) hazardous drinkers, 3) harmful alcohol use), and 4) alcohol dependence [32, 33]. For ease of analysis, we dichotomized the categories in 1) low-risk drinker and 2) high-risk drinkers (which combined the three AUDIT categories: hazardous drinkers, harmful drinkers, and harmful alcohol use). Furthermore, we assessed heavy episodic drinking using the third item of the AUDIT. This item measures heavy episodic drinking or having six or more drinks on one occasion on a scale ranging from never to daily or almost daily. Participants were classified as “ever” heavy episodic drinkers (those who selected “less than monthly or more frequently) or “never” heavy episodic drinkers [34].

Cigarette smoking: Participants were categorized as: 1) non-smokers, 2) previous smokers, and 3) current smokers based on their questionnaire responses. Participants who reported currently smoking were asked to estimate the average number of cigarettes they consume on daily basis.

Physical exercise: In this study, physical exercise was defined as moderate-intensity activities (sport, fitness, or recreational activities that require moderate physical effort and cause small increases in breathing or heart rate) or vigorous-intensity activities (sport, fitness, or recreational activities that require hard physical effort and cause large increases in breathing or heart rate) for at least 10 continuous minutes during free time. Participants were specifically asked whether or not they were currently engaged in: 1) any moderate-intensity and 2) vigorous-intensity activities (response options included yes or no). Participants who responded “yes” were asked to provide their weekly frequency of physical exercise. We created the variable “currently engaged in physical exercise”, to distinguish participants who reported physical exercise (moderate-intensity or vigorous-intensity) from those who did not. This was used as the outcome variable for physical exercise.

### Ethics statement

The study was approved by the Chiang Mai University Research Institute for Health Sciences Human Experimentation Committee (Certificate of Ethical Clearance No.39/2015). Prior to study enrollment, participants were educated about the study’s objectives; the role of participants; and their rights, which included answering or not answering any question during the interview. All participants provided written informed consent, and were paid 200 Baht (~6 USD) for the cost of transportation and time.

### Statistical analysis

The analysis was performed using SPSS 17 (PASW) for Windows (SPSS Inc., Chicago, Illinois, USA). Univariate analysis was conducted to obtain descriptive statistics of all the variables. Univariate and multivariate logistic regressions were performed to obtain both unadjusted (OR) and adjusted odds ratios (AOR), and 95% confidence intervals (CI) of factors associated with the main outcomes. For alcohol use, we predicted the odds of “drunk any alcohol in the past 12 months” versus “no”. For tobacco smoking, we modeled the odds of being a “current smoker” versus “not current smoker (which combined non-smokers and previous smokers). Lastly, for physical exercise, we predicted the odds of “currently engaged in physical exercise of moderate and/or vigorous-intensity” versus “no”. The multivariate logistic regression models included variables that had *p* ≤ 0.10 at the bivariate analysis and variables we considered epidemiologically important. We did not include “waist circumference” and “living with spouse” in the same model because of their multicollinearity respectively with “BMI” and “marital status”.

## Results

### Demographics

We recruited a total of 364 HIV-infected participants. More than half were female (57.1%), and between 50–59 years old (67.3%). Most participants had at least completed primary school education (87.6%), were employed (79.4%), were Buddhist (96.7%), and lived with at least 2 family members (82.4%). A sizeable proportion of the participants lived in households with a monthly income less than 5,001 baht (1 USD = 35 THB) (44.2%), and perceived their household income as insufficient (45.3%). Nearly half of the participants (49.7%) had a BMI within the normal range.

Most of the participants reported being HIV positive (85.1%) and were on ART (79.0%) for more than 5 years. The majority were diagnosed with HIV before they were 50 years old (64.2%), and had a plasma viral load of 50 copies/mL or less (98.3%) (Table 1).

**Table 1:** Socio-demographic and clinical characteristics of HIV-infected and non-infected older adults in Chiang Mai, Thailand.

	N (364)	%
**Gender**		
Male	156	42.9
Female	208	57.1
**Age**		
50–54 years	121	33.2
55–59 years	124	34.1
60–64 years	76	20.9
≥ 65 years	43	11.8
Mean (SD)	57.8 (5.6)	
**Education**		
Never attended school	45	12.4
Primary school	264	72.5
Secondary school or higher	55	15.1
**Occupation**		
Unemployed	75	20.6
Employed	289	79.4
**Marital Status**		
Married	164	45.1
Single/Widowed/Divorced	200	54.9
**Religion**		
Buddhism	352	96.7
Christianity	10	2.7
Islam	2	0.5
**Current number of family members**		
≥ 2 persons	300	82.4
Alone	64	17.6
**Live with spouse**		
No	189	51.9
Yes	175	48.1
**Live with children**		
No	208	57.1
Yes	156	42.9
**Household income (Baht/month)**		
≤ 5,000	161	44.2
5,001–20,000	157	43.1
>20,000	46	12.6
**Perceived sufficiency of household income**		
Sufficient /savings	71	19.5
Sufficient /no savings	128)	35.2
Insufficient	165	45.3
**Waist circumference**^**#**^		
Below standard	253	69.5
Above standard	111	30.5
**BMI**		
< 18.5	77	21.2
18.5–22.9	181	49.7
≥ 23	106	29.1
**Age at HIV positive diagnosis**		
Before 50 years old	233	64.0
After 50 years old	130	35.7
Missing	1	0.3
**Ever had an opportunistic infection**		
Yes	107	29.4
No	257	70.6
**ARV treatment**		
Yes	362	99.5
No	2	0.5
**Plasma viral load**		
0–50 copies/mL	352	98.3
> 50 copies/mL	6	0.8
Missing	6	0.8
**Years taking ARV**		
0–5 years	76	21.0
6–10 years	168	46.4
> 11 years	118	32.6

BMI: Body Mass Index; ARV: antiretroviral drug; SD: Standard Deviation

# Waist circumference: below standard (Male < 90 cm; female < 80 cm); above standard (Male ≥ 90 cm; female ≥ 80 cm)

### Behavioral characteristics of participants

A total of 68 participants reported drinking alcohol in the past year, among whom, 15 (22.1%) and 16 (23.5%) participants respectively reported a history of high-risk drinking and heavy episodic drinking. A significantly higher proportion of participants reported having ever drunk alcohol were male than female (73.5% versus 26.5%, *p*< 0.001).

Fifteen percent of our participants were current smokers and 22% were previous smokers. A significantly higher proportion of current smokers were male than female (72.7% versus 27.3%, *p*< 0.001).

A substantial proportion (59.1%) of participants reported being currently engaged in physical activities in their free time. Moderate-intensity physical exercise was the most reported type of exercise (54.4%), followed by vigorous-intensity physical exercise (9.6%). There was no statistically significant difference between male and female participants in terms of physical exercise (44.2% versus 55.8%; *p* = 0.538) (Table 2).

**Table 2:** Behavioral characteristics of HIV-infected older adults in Chiang Mai, Thailand.

	N	%
**Drunk any alcohol in the past year**		
No	296 (81.3)	81.3
Yes	68 (18.7)	18.7
**AUDIT (N = 170)**		
Low risk drinker	53 (77.9)	77.9
High risk drinker	15 (22.1)	22.1
**Heavy episodic drinking**		
Never	52 (76.5)	76.5
Ever	16 (23.5)	23.5
**Smoking behavior**		
Non smoker	229 (62.9)	62.9
Previous smoker (quit > 3 months)	80 (22.0)	22.0
Current smoker	55 (15.1)	15.1
**Number of cigarettes smoked average per day (N = 116)**		
Less than 1–1 smoke occasionally	2 (3.8)	3.8
2–5 cigarettes per day	41 (78.8)	78.8
>5 cigarettes per day	9 (17.3)	17.3
**Engage in physical exercise** ^**#**^		
No	149 (40.9)	40.9
Yes	215 (59.1)	59.1
**Currently doing vigorous-intensity exercises**		
No	329 (90.4)	90.4
Yes	35 (9.6)	9.6
**If you do vigorous-intensity exercises, how often do you do them? (N = 35)**		
1–2 days a week	11 (31.4)	31.4
3–5 days a week	11 (31.4)	31.4
>5 days a week	13 (37.4)	37.4
**Currently doing moderate-intensity exercises**		
No	166 (45.6)	45.6
Yes	198 (54.4)	54.4
**If you do moderate-intensity exercises, how often do you do them? (N = 198)**		
1–2 days a week	42 (21.2)	21.2
3–5 days a week	65 (32.8)	32.8
>5 days a week	91 (46.0)	46.0

AUDIT: The Alcohol Use Disorders Identification Test

#: includes participants who reported being engaged in at least one of type of physical exercise (moderate-intensity or vigorous-intensity, or both)

### Factors associated with socio-behavioral characteristics of participants among HIV-infected participants

The current report includes results from the multivariate analysis (Table 3). Bivariate associations with behavioral characteristics are provided in a supplemental file (S1 Table). The analysis revealed that more male participants than females reported drinking alcohol in the past year (AOR, 4.66; CI, 2.28–9.49; *P*<0.001) and were current smokers (AOR, 13.41; CI, 7.23–24.87; *p*<0.001). However, there was no difference between males and females in terms of physical activity.

**Table 3:** Correlates of alcohol use, tobacco smoking, and physical exercise among OALHIV participants.

	Adjusted Odds Ratio (95%CI)		
	Alcohol drinking in the past year	Currently smoking	Currently doing physical exercises
**Gender**			
Female	1.00	1.00	1.00
Male	4.66 (2.28–9.49)^*†*^	13.41 (7.23–24.87)^*†*^	1.20 (0.71–2.02)
**Age**			
50–54 years	1.00	1.00	1.00
55–59 years	0.53 (0.26–1.07)^φ^	0.96 (0.49–1.87)	1. 76 (1.00–3.08)*
60–64 years	0.40 (0.14–1.14)^φ^	0.75 (0.30–1.89)	1.46 (0.69–3.08)
≥ 65 years	0.34 (0.08–1.42)	0.78 (0.24–2.54)	1.09 (0.41–2.94)
**Education**			
Never attended school	0.53 (0.13–2.05)	2.15 (0.74–6.20)	0.22 (0.08–0.55)*
Primary school	0.67 (0.28–1.58)	0.82 (0.36–1.85)	0.53 (0.26–1.08) ^φ^
Secondary school or higher	1.00	1.00	1.00
**Occupation**			
Unemployed	1.00	1.00	1.00
Employed	2.56 (0.90–7.30) ^φ^	0.73 (0.36–1.49)	1.41 (0.78–2.53)
**Marital Status**			
Married	1.00	1.00	1.00
Single/Widowed/Divorced	1.57 (0.83–2.95)	1.62 (0.92–2.86) ^φ^	0.74 (0.46–1.21)
**Household income (Baht/month)**			
≤ 5,000	5.34 (1.28–22.25)*	2.07 (0.73–5.89)	1.19 (0.52–2.71)
5,001–20,000	4.66 (1.21–17.88)*	1.91 (0.73–5.03)	1.09 (0.51–2.34)
>20,000	1.00	1.00	1.00
**Family financial status**			
Sufficient /savings	1.05 (0.44–2.51)	0.36 (0.15–0.84)*	1.04 (0.53–2.02)
Sufficient /no savings	0.81 (0.41–1.61)	0.98 (0.53–1.81)	1.33 (0.80–2.21)
Insufficient	1.00	1.00	1.00
**Waist circumference**			
Below standard	1.00		1.00
Above standard	0.96 (0.44–2.11)		1.96 (1.15–3.34) *
**BMI**			
< 18.5		1.93 (0.90–4.14) ^φ^	
18.5–22.9		0.97 (0.51–1.85)	
≥ 23		1.00	
**Years on ARV treatment**			
0–5 years	1.00	1.00	1.00
6-10 years	0.86 (0.35–2.11)	0.96 (0.46–2.00)	0.99 (0.53–1.84)
>11 years	1.50 (0.56–4.03)	0.58 (0.25–1.35)	0.90 (0.45–1.80)
**Timing of HIV status**			
Before 50 years old	1.00	1.00	1.00
After 50 years old	0.96 (0.35–2.65)	0.82 (0.35–1.91)	1.00 (0.49–2.03)
**Ever had an opportunistic infection**			
Yes	1.00	1.00	1.00
No	1.21 (0.64–2.29)	1.06 (0.59–1.92)	1.04 (0.64–1.70)

* *P* value < 0.05.

** *P* value < 0.01.

^*†*^*P* value <0.001.

^φ^ *P* value <0.10.

# Waist circumference: below standard (Male < 90 cm; female < 80 cm); above standard (Male ≥ 90 cm; female ≥ 80 cm).

There was a relation between alcohol use and household income. Participants with a monthly household income ≤ 5,000 Baht and those with incomes ranging from > 5,000 to 20,000 baht were more likely to report having drunk alcohol in the past year (household income of ≤ 5,000 baht versus > 20,000 baht: AOR, 5.34; CI, 1.28–22.25; *p* = 0.021; household income of > 5,000–20, 000 baht versus >20,000 baht: AOR, 4.66; CI, 1.21–17.88; *p* = 0.025).

We also found that participants who never attended school were less likely to engage in physical exercises compared to those who had secondary or higher education levels (AOR, 0.22; CI, 0.08–0.55; *p* = 0.001). In addition, participants with a waist circumference above the normal standards were more likely to report being currently engaged in physical exercises (AOR, 1.96; CI, 1.15–3.34; *p* = 0.013).

## Discussion

This study describes and reports correlates of major health behaviors including alcohol use, smoking, and physical exercise among OALHIVs in Thailand. These socio-behavioral risk factors are increasingly associated with poor health outcomes in HIV-infected individuals in high-income countries, but have remained understudied in LMICs [13, 14, 35, 36]. We are not aware of previous studies in LMICs that have specifically focused on OALHIVs to explore alcohol use, smoking and physical activity. The fact that the health impacts of alcohol use, smoking and physical inactivity might be even more pronounced for OALHIVs, and that this population is expected to increase, highlights the relevance of focusing attention on socio-behavioral risk factors.

In our study, nearly one-fifth of the participants reported having drunk alcohol in the past 12 months, of which 23.5% experienced heavy episodic drinking and 22.1% were classified as high-risk drinkers. In a large survey conducted in three provinces in Northern Thailand including Chiang Mai, 25% of adults aged 50 years old were daily alcohol users [30]. The prevalence of “drank any alcohol in the past 12 months” in the Thailand NHES IV was 24.5% for people aged 60 years and older and 29.4% for those aged at least 45 years. However, the prevalence of heavy episodic drinking in the NHES IV, which was a national representative probability sampled survey, was much lower than that documented in our study, 2% and 4.9% for people aged at least 60 years and for those aged at least 45 years respectively [31].

Smoking is associated with increased risk of non-AIDS related mortality among HIV-infected individuals [13, 14], and its prevalence in people living with HIV infection was shown to be generally higher than that of the general population in the settings where the studies were conducted [9, 13, 37, 38]. The proportion of participants who were current smokers in our study was similar to the one reported in the Thai NHES IV in the general population [31]. A recent study found that HIV infection was not only an independent risk factor for smoking but = also decreased the likelihood of quitting smoking [39]. Several tobacco cessation programs have been shown to be effective with HIV-infected individuals, but most were limited by short follow-up, a non-randomized design, and the use cognitive behavioral strategies, which are hard to implement and scale-up within the care models in many LMICs [40–43]. HIV-infected individuals remain a high-risk group in need of targeted HIV smoking cessation interventions, deliverable within the routine care models [39, 44]. We also found that a significant proportion of our participants engaged in physical activity. Approximately 59% reported having physical activity of moderate or vigorous intensity at least 1–2 days a week. This proportion is much larger than that reported in the general population in Thailand, 21.4% and 23.7%, respectively for people aged at least 60 years and for those aged at least 45 years [31].

This study has also brought to light some of the risk factors associated with alcohol use, smoking, and physical activity among OALHIVs in Thailand. We found that male OALHIVs were more likely to report alcohol use and to be current smokers than their female counterparts. The difference in drinking and smoking behaviors in males and females is extensively documented [45–50]. In the context of our study, the gender difference likely reflects social and cultural norms that condone men’s smoking and drinking but disapprove of these behaviors in women. Such traditional norms tend to prevail among the older generation such as the participants in our study [49, 51, 52]. On the other hand, there was no statistically significant difference between male and female OALHIVs in terms of physical exercise. There is a remarkable scarcity of literature examining gender differentials in physical activity among HIV-infected individuals, particularly in OALHIVs. The only study we are aware of is a recently published study that found that HIV-infected men and women aged 51 years and older were similar in terms of frequency, average intensity and average hours of exercise. However, the study was limited by its small sample size of 27 men and 18 women aged 51 years and older [53].

We also found that the likelihood engaging in exercise decreased with the level of education among HIV-infected participants. Physical activity should be encouraged among OALHIVs with particular emphasis given to those with lower educational attainment.

Previous studies conducted among HIV-infected individuals [54–57], and in the general population [58–60] have documented higher prevalence of alcohol use/disorders and smoking in disadvantaged groups. In this study, lower household income was associated with increased odds of alcohol use in the past year. A previous review and critique of the literature highlighted the limited evidence of interventions to reduce alcohol use among HIV-infected individuals [61]. Effective interventions targeting socially disadvantaged groups in the general population are equally scarce. An ongoing trial is testing the effectiveness of mobile phone text messages to reduce binge drinking among disadvantaged men in Scotland [62]. Similar trials should be conducted in HIV-infected individuals given the ubiquitous nature of mobile phones even in disadvantaged groups. The fact that OALHIVs with higher waist circumference were more likely to be currently engaged in physical exercises suggests that overweight/obese individuals might be more health-conscious and aware of the adverse outcomes associated with overweight and/or obesity.

The interpretation of our findings should be examined in the light of study limitations. The cross-sectional design does not allow for drawing causal inferences from the documented associations. Caution is warranted in generalizing the findings of this study. Our participants were recruited from districts hospitals serving a large population of HIV-infected individuals in Chiang Mai province. Because we did not apply random sampling, our results may not be representative of population of OALHIVs in Chiang Mai.

## Conclusions

A substantial proportion of OALHIVs in our study were current smokers and reported alcohol drinking, with a particularly higher proportion of heavy episodic drinking than that documented in similar age groups in the general population. Male gender was a strong predictor of having drunk alcohol in the past 12 months and being a current smoker, while low socio-economic status (income and education) was a predictor of lack of physical exercise and alcohol use. Hence, we recommend that safe and effective interventions should be developed to deter smoking and alcohol use, and to promote physical exercises in OALHIVs, with a special focus on males and those of lower socio-economic status.

## Supporting information

S1 Table Bivariate factors associated with socio-behavioral risk factors. (DOCX)

S1 File Questionnaire Thai version. (DOC)

S2 File Questionnaire English version. (DOCX)

S3 File Medical record and clinical examination information. (PDF)

S1 Dataset Dataset of the study. (SAV)
