# Correction: Socio-behavioral risk factors among older adults living with HIV in Thailand

**DOI:** 10.1371/journal.pone.0192818

**Published:** 2018-02-08

**Authors:** Patou Masika Musumari, Arunrat Tangmunkongvorakul, Kriengkrai Srithanaviboonchai, Mitchell D. Feldman, Wathee Sitthi, Kittipan Rerkasem, Teeranee Techasrivichien, S. Pilar Suguimoto, Masako Ono-Kihara, Masahiro Kihara

The third author’s name is misspelled. The correct name is: Kriengkrai Srithanaviboonchai.

The correct citation is: Musumari PM, Tangmunkongvorakul A, Srithanaviboonchai K, Feldman MD, Sitthi W, Rerkasem K, et al. (2017) Socio-behavioral risk factors among older adults living with HIV in Thailand. PLoS ONE 12(11): e0188088. https://doi.org/10.1371/journal.pone.0188088
