# Supplementary material for: Socio-behavioral risk factors among older adults living with HIV in Thailand
Source: PLoS One. 2017 Nov 14;12(11):e0188088. doi: 10.1371/journal.pone.0188088 (PMC5685602; doi:10.1371/journal.pone.0188088)
Supplement: S1 Table — (DOCX) [file pone.0188088.s001.docx]

**S1 Table.** Bivariate factors associated with alcohol use, tobacco smoking, and physical exercise among HIV-infected older adults

|  | Odds Ratio (95%CI) | | |
| --- | --- | --- | --- |
|  | Ever drank alcohol in the past year | Currently smoking | Currently engaged in physical exercises |
| **Gender** |  |  |  |
| Female | 1.00 | 1.00 | 1.00 |
| Male | 4.97 (2.76-8.97)*^†^* | 4.43 (2.34-8.38)*^†^* | 1.14 (0.74-1.74) |
| **Age** |  |  |  |
| 50-54 years | 1.00 | 1.00 | 1.00 |
| 55-59 years | 0.49 (0.26-0.91) ^*^ | 0.86 (0.45-1.66) | 1.73 (1.03-2.91)^*^ |
| 60-64 years | 0.34 (0.15-0.76) ^**^ | 0.57 (0.24-1.31) | 1.36 (0.76-2.44) |
| ≥ 65 years | 0.33 (0.12-0.92)^*^ | 0.20 (0.04-0.92) | 1.02 (0.51-2.05) |
| **Education** |  |  |  |
| Never attended school | 0.39 (0.11-1.32) | 1.26 (0.40-3.91) | 0.22 (0.09-0.53)^**^ |
| Primary school | 1.00 (0.48-2.07) | 1.26 (0.53-2.98) | 0.55 (0.29-1.06) ^φ^ |
| Secondary school or higher | 1.00 | 1.00 | 1.00 |
| **Occupation** |  |  |  |
| No employment | 1.00 | 1.00 | 1.00 |
| Currently have a job | 3.90 (1.51-10.08) ^**^ | 1.38 (0.64-2.98) | 1.43 (0.86-2.39) |
| **Marital Status** |  |  |  |
| Married | 1.00 | 1.00 | 1.00 |
| Single/Widowed/Divorced | 0.43 (0.25-0.74)^**^ | 0.63 (0.35-1.13) | 1.19 (0.78-1.81) |
| **Religion** |  |  |  |
| Buddhism | 1.00 | 1.00 | 1.00 |
| Christianity | 1.89 (0.47-7.51) | 0.62 (0.07-5.05) | 0.28 (0.07-1.12) ^φ^ |
| Islam | N/A | 5.64 (0.34-91.58) | 0.66 (0.04-10.77) |
| **Current family member** |  |  |  |
| ≥ 2 persons | 1.00 | 1.00 | 1.00 |
| Alone | 1.27 (0.65-2.47) | 1.20 (0.58-2.49) | 1.01 (0.58-1.75) |
| **Live with spouse** |  |  |  |
| No | 1.00 | 1.00 | 1.00 |
| Yes | 1.98 (1.15-3.39)^*^ | 1.48 (0.83-2.63) | 0.74 (0.49-1.13) |
| **Live with children** |  |  |  |
| No | 1.00 | 1.00 | 1.00 |
| Yes | 0.67 (0.39-1.17) | 0.66 (0.36-1.20) | 1.25 (0.82-1.91) |
| **Household income (Baht/month)** |  |  |  |
| ≤ 5,000 | 3.28 (0.95-11.29) | 10.30 (1.36-77.75)^*^ | 0.72 (0.36-1.44) |
| 5,001 – 20,000 | 4.11 (1.20-14.05)^*^ | 8.1 (1.06-61.75)^*^ | 0.75 (0.38-1.49) |
| >20,000 | 1.00 | 1.00 | 1.00 |
| **Family financial status** |  |  |  |
| Sufficient /savings | 1.06 (0.52-2.14) | 0.35 (0.13-0.96) | 1.24 (0.70-2.20) |
| Sufficient /no savings | 0.94 (0.52-1.72) | 0.92 (0.49-1.70) | 1.40 (0.87-2.24) |
| Insufficient | 1.00 | 1.00 | 1.00 |
| **BMI** |  |  |  |
| < 18.5 | 1.49 (0.71-3.10) | 1.85 (1.01-3.40)^*^ | 0.59 (0.32-1.07) ^φ^ |
| 18.5 – 22.9 | 1.05 (0.55-1.98) | 1.18 (0.71-1.97) | 0.96 (0.58-1.57) |
| ≥ 23 | 1.00 | 1.00 | 1.00 |
| **Waist circumference** |  |  |  |
| Below standard | 1.00 | 1.00 | 1.00 |
| Above standard | 0.42 (0.21-0.83)^*^ | 0.52 (0.25-1.05) ^φ^ | 1.78 (1.11-2.85)^*^ |
| **Had started ARV treatment** |  |  |  |
| 0-5 years | 1.00 | 1.00 | 1.00 |
| - 1. years | 1.20 (0.58-2.50) | 1.49 (0.71-3.14) | 1.03 (0.59-1.80) |
| >11 years | 1.43 (0.67-3.06) | 0.48 (0.19-1.24) | 0.93 (0.52-1.68) |
| **Timing of HIV status** |  |  |  |
| Before 50 years old | 1.00 | 1.00 | 1.00 |
| After 50 years old | 0.58 (0.32-1.05) ^φ^ | 0.77 (0.41-1.43) | 1.01 (0.65-1.57) |
| **Ever had opportunistic infection** |  |  |  |
| Yes | 1.00 | 1.00 | 1.00 |
| No | 1.29 (0.73-2.26) | 0.79 (0.41-1.52) | 1.04 (0.66-1.65) |

^*^ *P* value < 0.05; ^**^ *P* value < 0.01; *^†^P* value <0.001; ^φ^ *P* value <0.10; CI: confidence interval
