# Supplementary material for: Socio-behavioral risk factors among older adults living with HIV in Thailand
Source: PLoS One. 2017 Nov 14;12(11):e0188088. doi: 10.1371/journal.pone.0188088 (PMC5685602; doi:10.1371/journal.pone.0188088)
Supplement: S1 File — (DOC) [file pone.0188088.s002.doc]

**แบบสัมภาษณ์อาสาสมัคร**

รหัสอาสาสมัคร…………………..…………..……………………….…………. รหัสผู้สัมภาษณ์………………………………..……….

วันที่สัมภาษณ์ วันที่ ………เดือน……………..……..พ.ศ.2558 เริ่มสัมภาษณ์เวลา ……………………….….……. น.

คำชี้แจง กรุณาตอบคำถามตรงตามข้อเท็จจริง หากมีข้อสงสัยประการใดสามารถสอบถามพนักงานสัมภาษณ์ได้ตลอดเวลา

**ส่วนที่ 1 ข้อมูลทั่วไป**

1. เพศ 🗖1.ชาย 🗖2. หญิง
2. ขณะนี้ท่านอายุ .............ปี
3. ระดับการศึกษาสูงสุดของท่านคือ

🗖1. ไม่ได้เรียน 🗖2. ประถมศึกษา 🗖3. มัธยมศึกษาตอนต้น

🗖4. มัธยมศึกษาตอนปลาย / ปวช. 🗖5. ปวส. / อนุปริญญา

🗖6. ปริญญาตรี หรือสูงกว่า 🗖99. อื่นๆ ระบุ…………………….……..….

1. ปัจจุบันท่านประกอบอาชีพใด (เลือกตอบข้อเดียวที่เป็นอาชีพหลัก)

🗖1. ประกอบธุรกิจส่วนตัวโดยมีลูกจ้าง

🗖2. ประกอบธุรกิจส่วนตัวโดยไม่มีลูกจ้าง

🗖3. รับจ้างทั่วไป

🗖4. ลูกจ้างบริษัทเอกชน

🗖5. งานราชการ (ข้าราชการ ลูกจ้างประจำ ลูกจ้างชั่วคราว)

🗖6. งานรัฐวิสาหกิจ (พนักงาน ลูกจ้าง)

🗖7. ช่วยธุรกิจในครัวเรือนโดยไม่ได้รับค่าจ้าง

🗖8. การร่วมกลุ่ม (สหกรณ์ เครือข่ายขายตรง)

🗖9. เกษตรกร

🗖10. ไม่ได้ทำงาน (เกษียณ แม่บ้าน)

🗖99. อื่น ๆ โปรดระบุ……………………..

1. สถานภาพการสมรสของท่าน

🗖1. โสด 🗖2. มีคู่และอยู่ด้วยกัน

🗖3. มีคู่แต่แยกกันอยู่ 🗖4. หย่า / หม้าย

1. ท่านนับถือศาสนาใด

🗖1. พุทธ 🗖2. คริสต์

🗖3. อิสลาม 🗖4. ไม่ได้นับถือศาสนา

🗖99. อื่น ๆ โปรดระบุ……………………..

1. บ้านที่ท่านอยู่ในปัจจุบัน มีสมาชิกอยู่ด้วยกันทั้งหมด.......................คน ประกอบด้วยใครบ้าง (ตอบได้หลายข้อ)

🗖1. สามี / ภรรยา 🗖2. ลูก

🗖3. หลาน (ลูกของลูก) 🗖4. ญาติพี่น้อง

🗖99. อื่น ๆ โปรดระบุ......................................................................

1. ปัจจุบัน**ครอบครัวของท่าน**มีรายได้รวมกันเท่าใด (เลือกตอบข้อใดข้อหนึ่ง)

8.1 เดือนละประมาณ …………….…. บาท หรือ

8.2 ปีละประมาณ………………………. บาท (กรณีทำเกษตรกรรม)

1. ปัจจุบันรายได้เมื่อเทียบกับรายจ่ายของครอบครัวเป็นอย่างไร

🗖1. มีเหลือเก็บ 🗖2. พอดีกับค่าใช้จ่าย 🗖3. ไม่พอกับค่าใช้จ่าย

**ส่วนที่ 2 พฤติกรรมสุขภาพ**

1. โดยปกติในเวลาว่างจากการทำงาน ท่านมีกิจกรรมเล่นกีฬา การออกกำลังกาย หรือกิจกรรมยามว่าง **อย่างหนักจนทำให้หายใจแรงขึ้นหรือหัวใจเต้นเร็วขึ้นมาก** เป็นเวลาตั้งแต่ 10 นาทีขึ้นไปในแต่ละครั้ง (เช่น เต้นแอโรบิค วิ่ง หรือเล่นกีฬาอย่างหนัก เช่น ฟุตบอล) หรือไม่

🗖1. ใช่ 🗖2. ไม่ใช่ (ข้ามไปตอบข้อ 12)

1. ถ้าใช่ ปกติท่านเล่นกีฬาออกกำลังกายหรือกิจกรรมยามว่าง**อย่างหนัก** เป็นเวลาอย่างน้อย10 นาที ขึ้นไปในแต่ละครั้ง เป็นจำนวนกี่วันต่อสัปดาห์

............วันต่อสัปดาห์

1. โดยปกติในเวลาว่างจากการทำงาน ส่วนใหญ่ท่านมีกิจกรรมเล่นกีฬา ออกกำลังกายหรือมีกิจกรรมอย่าง**ปานกลาง** เป็นเวลาตั้งแต่ 10 นาทีขึ้นไปในแต่ละครั้ง (เช่น เดินเร็ว ขี่จักรยาน เล่นวอลเล่ย์บอล ว่ายน้ำ) หรือไม่

🗖1. ใช่ 🗖2. ไม่ใช่ (ข้ามไปตอบข้อ 14 )

1. ถ้าใช่ ปกติท่าน เล่นกีฬา ออกกำลังกายหรือมีกิจกรรมอย่าง เป็นเวลาอย่างน้อย 10 นาทีขึ้นไปในแต่ละครั้งเป็นจำนวนกี่วันต่อสัปดาห์

................ วันต่อสัปดาห์

1. พฤติกรรมการสูบบุหรี่ของท่านเข้าได้กับข้อใดต่อไปนี้

🗖1. ไม่เคยสูบบุหรี่ (ข้ามไปตอบข้อ 16)

🗖2. เคยแค่ลองสูบ แต่ปัจจุบันไม่ได้สูบ (ข้ามไปตอบข้อ 16)

🗖3. เคยสูบแต่เลิกแล้ว โดยเลิกสูบมาเป็นเวลา ……............... เดือน (ข้ามไปตอบข้อ 16)

🗖4. ปัจจุบันยังสูบบุหรี่อยู่

1. ปัจจุบันท่านสูบบุหรี่เฉลี่ยวันละ ...................... มวน

**หากอาสาสมัครไม่ดื่มสุรา ให้ข้ามไปถามคำถามส่วนที่ 3 คุณภาพชีวิต (ข้อ 26)**

**คำชี้แจง :** คำถามแต่ละข้อต่อไปนี้จะถามถึงประสบการณ์การดื่มสุราในรอบ 1 ปีที่ผ่านมา โดยสุรา หมายถึงเครื่องดื่มที่มีแอลกอฮอล์ทุกชนิด ได้แก่ เบียร์ เหล้า สาโท กระแช่ วิสกี้ สปายไวน์ เป็นต้น

| ข้อคำถาม | 0 | 1 | 2 | 3 | 4 |
| --- | --- | --- | --- | --- | --- |
| 1. คุณดื่มสุราบ่อยเพียงไร | ไม่เคยเลย | เดือนละครั้งหรือน้อยกว่า | 2-4 ครั้ง  ต่อเดือน | 2-3 ครั้ง  ต่อสัปดาห์ | 4 ครั้งขึ้นไป  ต่อสัปดาห์ |
| 1. (เลือกตอบเพียงข้อเดียว)   เวลาที่คุณดื่มสุรา โดยทั่วไปแล้วคุณดื่มประมาณเท่าไรต่อวัน **หรือ** | 1-2  ดื่มมาตรฐาน | 3-4  ดื่มมาตรฐาน | 5-6  ดื่มมาตรฐาน | 7-9  ดื่มมาตรฐาน | ตั้งแต่ 10  ดื่มมาตรฐาน  ขึ้นไป |
| ถ้าโดยทั่วไปดื่มเบียร์ ประมาณเท่าไร ต่อวัน  **หรือ** | 1-1.5 กระป๋อง/  1/2-3/4 ขวด | 2-3 กระป๋อง/  1-1.5 ขวด | 3.5-4กระป๋อง/  2 ขวด | 4.5-7 กระป๋อง/  3-4 ขวด | 7 กระป๋อง/  4 ขวดขึ้นไป |
| ถ้าโดยทั่วไปดื่มเหล้า (เช่น แม่โขง หงส์ทอง หงส์ทิพย์ เหล้าขาว 40 ดีกรี) ประมาณเท่าไรต่อวัน | 2-3 ฝา | 1/4 แบน | 1/2 แบน | 3/4 แบน | 1 แบนขึ้นไป |
| 1. บ่อยครั้งเพียงไรที่คุณดื่มตั้งแต่ 6 ดื่มมาตรฐานขึ้นไป หรือเบียร์ 4 กระป๋องหรือ 2 ขวดใหญ่ ขึ้นไป หรือเหล้าวิสกี้ 3 เป๊กขึ้นไป | ไม่เคยเลย | น้อยกว่า  เดือนละครั้ง | เดือนละครั้ง | สัปดาห์  ละครั้ง | ทุกวัน หรือ  เกือบทุกวัน |
| 1. บ่อยครั้งเพียงไรที่คุณพบว่าคุณไม่สามารถหยุดดื่มได้ หากคุณได้เริ่มดื่มไปแล้ว | ไม่เคยเลย | น้อยกว่า  เดือนละครั้ง | เดือนละครั้ง | สัปดาห์  ละครั้ง | ทุกวัน หรือ  เกือบทุกวัน |
| 1. บ่อยครั้งเพียงไรที่คุณไม่ได้ทำสิ่งที่คุณควรจะทำตามปกติ เพราะคุณมัวแต่ไปดื่มสุราเสีย | ไม่เคยเลย | น้อยกว่า  เดือนละครั้ง | เดือนละครั้ง | สัปดาห์  ละครั้ง | ทุกวัน หรือ  เกือบทุกวัน |
| 1. บ่อยครั้งเพียงไรที่คุณต้องดื่มสุราทันทีในตอนเช้า เพื่อจะได้ดำเนินชีวิตตามปกติ หรือถอนอาการเมาค้างจากการดื่มหนักในคืนที่ผ่านมา | ไม่เคยเลย | น้อยกว่า  เดือนละครั้ง | เดือนละครั้ง | สัปดาห์  ละครั้ง | ทุกวัน หรือ  เกือบทุกวัน |
| 1. บ่อยครั้งเพียงไรที่คุณรู้สึกไม่ดี โกรธ หรือเสียใจ เนื่องจากคุณได้ทำบางสิ่งบางอย่างลงไปขณะที่คุณดื่มสุราเข้าไป | ไม่เคยเลย | น้อยกว่า  เดือนละครั้ง | เดือนละครั้ง | สัปดาห์  ละครั้ง | ทุกวัน หรือ  เกือบทุกวัน |
| 1. บ่อยครั้งเพียงไรที่คุณไม่สามารถจำได้ว่าเกิดอะไรขึ้นในคืนที่ผ่านมา เพราะว่าคุณได้ดื่มสุราเข้าไป | ไม่เคยเลย | น้อยกว่า  เดือนละครั้ง | เดือนละครั้ง | สัปดาห์  ละครั้ง | ทุกวัน หรือ  เกือบทุกวัน |
| 1. ตัวคุณเองหรือคนอื่น เคยได้รับบาดเจ็บซึ่งเป็นผลจากการดื่มสุราของคุณหรือไม่ | ไม่เคยเลย |  | เคย แต่ไม่ได้  เกิดขึ้นในปีที่แล้ว |  | เคยเกิดขึ้นใน  ช่วงหนึ่งปีที่ผ่านมา |
| 1. เคยมีแพทย์ หรือบุคลากรทางการแพทย์หรือเพื่อนฝูงหรือญาติพี่น้องแสดงความเป็นห่วงเป็นใยต่อการดื่มสุราของคุณหรือไม่ | ไม่เคยเลย |  | เคย แต่ไม่ได้  เกิดขึ้นในปีที่แล้ว |  | เคยเกิดขึ้นใน  ช่วงหนึ่งปีที่ผ่านมา |

**ส่วนที่ 3 คุณภาพชีวิต**

1. โดยทั่วๆ ไป ท่านสามารถพูดได้ว่าสุขภาพของท่านอยู่ในระดับใด

🗖1. ดีเยี่ยม 🗖2. ดีมาก 🗖3. ดี 🗖4. พอใช้ 🗖5. ไม่ดี

1. ในช่วง 1 เดือนที่ผ่านมา ท่านมีอาการเจ็บปวดตามร่างกาย (เช่น ปวดหัว ปวดตามตัว ปวดข้อ ฯลฯ) มากน้อยเพียงใด

🗖1. ไม่มีเลย 🗖2. น้อยมาก 🗖3. น้อย 🗖4. ปานกลาง 🗖5. รุนแรง 🗖6. รุนแรงมาก

1. ในช่วง 1 เดือนที่ผ่านมา อาการเจ็บปวดที่รบกวนกิจกรรมตามปกติของท่านมีมากน้อยเพียงใด (กิจกรรมตามปกติ ทั้งการทำงานนอกบ้านและงานในบ้าน)

🗖1. ไม่มีเลย 🗖2. เล็กน้อย 🗖3. ปานกลาง 🗖4. ค่อนข้างมาก 🗖5. มากที่สุด

1. คำถามต่อไปนี้เกี่ยวกับกิจกรรมที่ท่านทำในแต่ละวัน ท่านคิดว่าสุขภาพของท่านในปัจจุบันเป็นปัญหา/อุปสรรคต่อการทำกิจกรรมตามปกติของท่านหรือไม่ ถ้าใช่ มากน้อยแค่ไหน

|  | ใช่,เป็นปัญหา/อุปสรรค  อย่างมาก  (1) | ใช่,เป็นปัญหา/อุปสรรค  เพียงเล็กน้อย  (2) | ไม่เลย  (3) |
| --- | --- | --- | --- |
| 29.1 กิจกรรมที่ใช้แรงมากที่ท่านสามารถทำได้ เช่น ขุดดิน ทำไร่ ทำสวน หรือเล่นกีฬาที่ต้องใช้แรงมาก ๆ เช่น ฟุตบอล |  |  |  |
| 29.2 กิจกรรมที่ใช้แรงปานกลาง ที่ท่านสามารถทำได้ เช่น การย้ายโต๊ะ, หรือหิ้วน้ำ |  |  |  |
| 29.3 การเดินขึ้นเนิน หรือเดินขึ้นตึก 2-3 ชั้น |  |  |  |
| 29.4 การก้ม ๆ เงย ๆ ยกของ หรือย่อตัว |  |  |  |
| 29.5 การเดินไกลประมาณ 1 ช่วงเสาไฟฟ้า |  |  |  |
| 29.6 แต่งตัว อาบน้ำ หรือการใช้ห้องน้ำ การกินอาหารได้ด้วยตนเอง |  |  |  |

1. ด้วยสุขภาพของท่าน ทำให้ท่านไม่สามารถทำงานประจำวันทุกประเภท (เช่น ทำงานบ้าน ไปไร่ ไปทุ่งนา) หรือไม่?

🗖1. ใช่ 🗖2. ไม่ใช่

1. ด้วยสุขภาพของท่าน ทำให้ท่านทำงานประจำวันบางประเภทได้น้อยลง หรือไปไร่นาได้เป็นบางวันหรือไม่?

🗖1. ใช่ 🗖2. ไม่ใช่

1. กรุณาเลือกเพียงคำตอบเดียวที่ท่านคิดว่าใกล้เคียงกับความรู้สึกของท่านมากที่สุด ในช่วง 1 เดือนที่ผ่านมา

| ในช่วง 1 เดือนที่ผ่านมา……… | ตลอด  เวลา  (1) | เกือบตลอดเวลา (2) | ค่อน  ข้างบ่อย  (3) | บางครั้ง  (4) | นานๆครั้ง  (5) | ไม่เลย  (6) |
| --- | --- | --- | --- | --- | --- | --- |
| 32.1 บ่อยแค่ไหนที่ สุขภาพของท่านเป็นปัญหา/อุปสรรคต่อการร่วมกิจกรรมทางสังคม (เช่น การเยี่ยมเพื่อนหรือญาติสนิท) |  |  |  |  |  |  |
| 32.2 บ่อยแค่ไหนที่……….. |  |  |  |  |  |  |
| 32.2.1 ท่านรู้สึกมีความสุข |  |  |  |  |  |  |
| 32.2.2 ท่านรู้สึกใจสงบ ใจนิ่ง มีสมาธิ |  |  |  |  |  |  |
| 32.2.3 ท่านรู้สึกเศร้า หดหู่ |  |  |  |  |  |  |
| 32.2.4 ท่านรู้สึกกระวนกระวายอยู่ไม่เป็นสุข |  |  |  |  |  |  |
| 32.2.5 ท่านรู้สึกแย่มากจนไม่มีอะไรที่ทำให้รู้สึกดีขึ้นได้ |  |  |  |  |  |  |
| 32.3 บ่อยแค่ไหนที่……….. |  |  |  |  |  |  |
| 32.3.1 ท่านรู้สึกแข็งแรง กระปรี้กระเปร่า สดชื่น |  |  |  |  |  |  |
| 32.3.2 ท่านมีแรงมากพอที่จะทำทุกสิ่งที่ท่านต้องการ |  |  |  |  |  |  |
| 32.3.3 ท่านรู้สึกเหนื่อยล้า หมดแรง |  |  |  |  |  |  |
| 32.3.4 ท่านรู้สึกอ่อนเพลีย |  |  |  |  |  |  |
| 32.3.5 ท่านรู้สึกหนักใจเนื่องจากปัญหาสุขภาพของท่าน |  |  |  |  |  |  |
| 32.3.6 ท่านรู้สึกท้อใจเนื่องจากปัญหาสุขภาพของท่าน |  |  |  |  |  |  |
| 32.3.7 ท่านรู้สึกหมดหวังเนื่องจากปัญหาสุขภาพของท่าน |  |  |  |  |  |  |
| 32.3.8 ท่านรู้สึกหวาดกลัวเนื่องจากสุขภาพของท่าน |  |  |  |  |  |  |
| 32.4 บ่อยแค่ไหนในช่วง 1 เดือนที่ผ่านมา…… |  |  |  |  |  |  |
| 32.4.1 ท่านมีความยุ่งยากในการทำอะไรบางอย่างที่ต้องหาเหตุผลและแก้ไขปัญหา เช่น การเรียงลำดับสิ่งที่จะทำในวันนี้ว่า วันนี้จะไปหาหมอเสร็จแล้วจะไปตลาด หรือจะไปเยี่ยมญาติก่อนดี |  |  |  |  |  |  |
| 32.4.2 ท่านลืมบางสิ่งที่เพิ่งเกิดขึ้น เช่น ลืมของที่วางไว้หรือลืมนัด |  |  |  |  |  |  |
| 32.4.3 ท่านมีปัญหาในการให้ความสนใจในกิจกรรมใดกิจกรรมหนึ่งเป็นเวลานานๆ หรือไม่ เช่น อ่านหนังสือพิมพ์ ดูทีวี ฟังวิทยุ |  |  |  |  |  |  |
| 32.4.4 ท่านมีปัญหาในการทำกิจกรรมที่เกี่ยวกับการใช้สมาธิและความคิดหรือไม่ เช่น ซ่อมรถ จักสาน เย็บผ้า แกะสลัก เขียนหนังสือ ทอผ้า |  |  |  |  |  |  |

1. ในช่วง 1 เดือนที่ผ่านมา รู้สึกว่าชีวิตของท่านเป็นอย่างไรบ้าง หมายถึงสิ่งต่างๆ ที่เกิดขึ้นกับชีวิตของท่านในทุกๆ ด้าน

🗖1. ดีมาก 🗖2. ดีพอใช้ 🗖3. ดี และแย่ พอๆ กัน 🗖4. ค่อนข้างแย่ 🗖5. แย่มาก และคงไม่แย่ไปกว่านี้แล้ว

1. ปัจจุบันนี้ ท่านคิดว่าสุขภาพร่างกายและสภาวะทางอารมณ์ของท่านอยู่ในระดับใด เมื่อเทียบกับ 1 เดือนก่อน

🗖1. ดีขึ้นมาก 🗖2. ดีขึ้นเล็กน้อย 🗖3. เหมือนเดิม 🗖4. แย่ลงเล็กน้อย 🗖5. แย่ลงมาก

**ส่วนที่ 4 สุขภาพ**

1. ท่านมีโรคประจำตัวดังต่อไปนี้หรือไม่

35.1 โรคความดันโลหิตสูง 🗖1. ใช่ 🗖2. ไม่ใช่

35.2 โรคไตวายเรื้อรัง 🗖1. ใช่ 🗖2. ไม่ใช่

35.3 โรคเบาหวาน 🗖1. ใช่ 🗖2. ไม่ใช่

35.4 ไขมันในเลือดสูง 🗖1. ใช่ 🗖2. ไม่ใช่

35.5 โรคประจำตัวอื่นๆ โปรดระบุ………………………………………………………………………………………………………..

*********************************************************************************

**สิ้นสุดการสัมภาษณ์เวลา ………….……..…. น.**
