# Supplementary material for: Socio-behavioral risk factors among older adults living with HIV in Thailand
Source: PLoS One. 2017 Nov 14;12(11):e0188088. doi: 10.1371/journal.pone.0188088 (PMC5685602; doi:10.1371/journal.pone.0188088)
Supplement: S2 File — (DOCX) [file pone.0188088.s003.docx]

**Questionnaire survey**

Participant ID…………………………………..… Interviewer code …………….…………….

Date of interview ………………………………… Time start to interview …………………….

*(dd-mm-yyyy Buddhist Era.)*

**Section 1: Socio-demographic information**

1. What is your gender?

__1. Male __2. Female

2. What was your age on your last birthday? _ _ Years old.

3. What is the highest level of education you have completed?

__1. Never attended school

__2. Primary school

__3. Secondary school

__4. High school/Vocational certificate

__5. High Vocational certificate/diploma

__6. Bachelor degree

_99. Other (Specify) ________________

4. What is your principal occupation? (Choose one answer)

__1. Business owner (with employee(s))

__2. Business owner (with no employee(s))

__3. Laborer/Employed for wages

__4. Private company employee

__5. Government sectors (Civil servant, permanent employee, temporary worker)

__6. State enterprise officer/employee

__7. Work in family business without pay

__8. Cooperative/Network marketing

__9. Farmer/Agriculturer

_10. Unemployed (Retired, housewife)

_99. Other (Specify) ________________

5. What is your marital status?

__1. Single

__2. Having partner (live together)

__3. Having partner (live apart)

__4. Widowed/Divorced

6. What is your religion?

__1. Buddhism __2. Christianity

__3. Islam __4. No religion

_99. Other (Specify) ________________

7. How many family members do you live with? _______ Persons

And who are they? *(You can choose more than one answer)*

__1. Spouse __2. Children

__3. Grandchildren __4. Relatives

_99. Other (Specify) ________________

8. How much is your current household income? (Choose to answer only one)

Monthly income_____________________ Baht

Yearly income ______________________ Baht (If doing agriculture)

9. What is the answer below that best describe your current household income when comparing with your household expenses?

__1. Sufficient, with savings

__2. Sufficient, with no savings

__3. Insufficient / not enough to spend

**Section 2: Health behaviour information**

*In answering the following questions,* ***'vigorous-intensity exercises'*** *are activities that require hard physical effort and cause large increases in breathing or heart rate****, 'moderate-intensity exercises'*** *are activities that require moderate physical effort and cause small increases in breathing or heart rate.*

10. Normally, when you have free time from the job, do you do any vigorous-intensity exercises, sports, fitness or recreational (leisure) activities that cause large increases in breathing or heart rate like [aerobic, running or football,] for at least 10 minutes continuously?

__1. Yes

__2. No  ***(IF No skip to Q12)***

11. In a typical week, on how many days do you do vigorous intensity sports, fitness or recreational (leisure) activities? ________ days a week

12. Normally, when you have free time from the job, do you do any moderate-intensity exercises, sports, fitness or recreational (leisure) activities that causes a small increase in breathing or heart rate such as brisk walking,(cycling, swimming, volleyball)for at least 10 minutes continuously?

__1. Yes

__2. No ***(IF No skip to Q14)***

13. In a typical week, on how many days do you do moderate-intensity sports, fitness or recreational (leisure) activities? ________ days a week

14. Which of these statements best describes your smoking behavior?

__1. Never smoked  ***(Skip to Q16)***

__2. Tried once or twice  ***(Skip to Q16)***

__3. Used to smoke but have quitted for ______months. ***(Skip to Q16)***

__4. Still smoke now

15. How many cigarettes do you smoke per day on average now ________cigarettes

***The following questions 16-25 only for those who have drunk alcohol.***

***Those who have never drunk alcohol please skip to Question 26***

Have you ever drunk alcohol in the past year?

__1. Yes

__2. No  ***(IF No skip to Q26)***

**The Alcohol Use Disorders Identification Test: AUDIT**

*Because alcohol use can affect your health and can interfere with certain medications and treatments, it is important that we ask some questions about your use of alcohol. Your answers will remain confidential so please be honest.*

*Place an X in one box that best describes your answer to each question.*

| **Questions** | **0** | **1** | **2** | **3** | **4** |
| --- | --- | --- | --- | --- | --- |
| 16. How often do you have a drink containing alcohol? | Never | Monthly  or less | 2-4 times  a month | 2-3 times  a week | 4 or more  times a week |
| **17.** **Please choose only one answer**  How many drinks containing alcohol do you have on a typical day when you are drinking?  **or** | 1-2  drinks | 3-4  drinks | 5-6  drinks | 7-9  drinks | 10 drinks  or more |
| How many drinks of beer (such as Singha, Heineken, Leo, Cheers, Tiger, and Chang) do you have on a typical day when you are drinking?  **or** | 1-1.5 cans/  ½ - ¾ bottle | 2-3 cans/  1-1.5 bottles | 3.5-4 cans/  2 bottles | 4.5-7 cans/  3-4 bottles | 7 cans/  4 bottles  or more |
| How many drinks of whiskey such as Mekhong, Hongthong, Hongthip, rice whiskey (containing 40 degrees of alcohol) do you have on a typical day when you are drinking? | 2-3 screw caps | ¼ flat bottle | ½ flat bottle | ¾ flat bottle | 1 flat bottle or more |
| 18. How often do you have six or more drinks on one occasion? | Never | Less than monthly | Monthly | Weekly | Daily or almost daily |
| 19. How often during the last year have you found that you were not able to stop drinking once you had started? | Never | Less than monthly | Monthly | Weekly | Daily or almost daily |
| 20. How often during the last year have you failed to do what was normally expected of you because of drinking? | Never | Less than monthly | Monthly | Weekly | Daily or almost daily |
| 21. How often during the last year have you needed a first drink in the morning to get yourself going after a heavy drinking session? | Never | Less than monthly | Monthly | Weekly | Daily or almost daily |
| 22. How often during the last year have you had a feeling of guilt or remorse after drinking? | Never | Less than monthly | Monthly | Weekly | Daily or almost daily |
| 23. How often during the last year have you been unable to remember what happened the night before because you had been drinking? | Never | Less than monthly | Monthly | Weekly | Daily or almost daily |
| 24. Have you or someone else been injured as a result of your drinking? | No |  | Yes, but not in the last year |  | Yes, during the last year |
| 25. Has a relative or friend or a doctor or another health worker been concerned about your drinking or suggested you cut down? | No |  | Yes, but not in the last year |  | Yes, during the last year |

***Remark:*** *1 flat bottle is around 300-375 ml.*

**Section 3: Quality of life**

*Please complete the following items after you completes the questionnaire or after you ascertain that is not possible.*

1. In general, would you say your health is:

__1. Excellent

__2. Very Good

__3. Good

__4. Fair

__5. Poor

27. How much **bodily** pain have you generally had during **the past 4 weeks?**

__1. None

__2. Very Mild

__3. Mild

__4. Moderate

__5. Severe

__6. Very Severe

28. During **the past 4 weeks**, how much did pain interfere with your normal work (or your normal activities, including work outside the home and housework)?

__1. Not at all

__2. A little bit

__3. Moderately

__4. Quite a bit

__5. Extremely

29. The following questions are about activities you might do during a typical day. Does your **health now limit you** in these activities? If so, how much?

| **No.** | **Questions** | **YES,**  **limited a lot**  1 | **YES,**  **limited a little**  2 | **NO,**  **not limited**  3 |
| --- | --- | --- | --- | --- |
| 29.1 | The kinds or amounts of **vigorous-intensity exercise** you can do, like lifting heavy objects, running or participating in strenuous sports. |  |  |  |
| 29.2 | The kinds or amounts of **moderate-intensity exercises** you can do, like moving a table, carrying groceries or bowling. |  |  |  |
| 29.3 | Walking uphill or climbing (a few flights of stairs). |  |  |  |
| 29.4 | Bending, lifting or stooping. |  |  |  |
| 29.5 | Walking one block. |  |  |  |
| 29.6 | Eating, dressing, bathing or using the toilet. |  |  |  |

30. Does **your health** keep you from working at a job, doing work around the house or going to school?

__1. Yes

__2. No

31. Have you been unable to do **certain kinds or amounts** of work, housework, or schoolwork because of your health?

__1. Yes

__2. No

32. For **each** of the following questions, please check the box for the **one** answer that comes **closest to** the way you have been feeling **during the past 4 weeks.**

| **No.** | **Questions** | **All of the**  **Time**  **1** | **Most of the Time**  **2** | **A Good**  **Bit of the Time**  **3** | **Some of the**  **Time**  **4** | **A Little of the Time**  **5** | **None of The Time**  **6** |
| --- | --- | --- | --- | --- | --- | --- | --- |
| 32.1. | How much of the time, during the past 4 weeks, has your **health limited your social activities** (like visiting with friends or close relatives)? |  |  |  |  |  |  |
| 32.2 | How much of the time, during the past 4 weeks: |  |  |  |  |  |  |
| 32.2.1 | Have you been a **happy person**? |  |  |  |  |  |  |
| 32.2.2 | Have you felt **calm and peaceful**? |  |  |  |  |  |  |
| 32.2.3 | Have you felt **downhearted and blue**? |  |  |  |  |  |  |
| 32.2.4 | Have you been a **very nervous person?** |  |  |  |  |  |  |
| 32.2.5 | Have you felt so **down in the dumps that nothing could cheer you up**? |  |  |  |  |  |  |
| 32.3 | How often during the  **past four weeks:** |  |  |  |  |  |  |
| 32.3.1 | Did you feel full of pep? |  |  |  |  |  |  |
| 32.3.2 | Did you have enough energy to do the things you wanted to do? |  |  |  |  |  |  |
| 32.3.3 | Did you feel worn out? |  |  |  |  |  |  |
| 32.3.4 | Did you feel tired? |  |  |  |  |  |  |
| 32.3.5 | Did you feel weighed down by your health problems? |  |  |  |  |  |  |
| 32.3.6 | Were you discouraged by your health problems? |  |  |  |  |  |  |
| 32.3.7 | Did you feel despair over your health problems? |  |  |  |  |  |  |
| 32.3.8 | Were you afraid because of your health? |  |  |  |  |  |  |
| 32.4 | How much of the time,  during the **past 4 weeks:** |  |  |  |  |  |  |
| 32.4.1 | Did you have difficulty reasoning and solving problems, for example, making plans, making decisions, and learning new things? |  |  |  |  |  |  |
| 32.4.2 | Did you forget things that happened recently, for example, where you put things and when you had appointments? |  |  |  |  |  |  |
| 32.4.3 | Did you have trouble keeping your attention on any activity for long? |  |  |  |  |  |  |
| 32.4.4 | Did you have difficulty doing activities involving concentration and thinking? |  |  |  |  |  |  |

33. Please check the box that best describes whether each of the following statements is true or false for you. (Check **one** box on **each** line)

| **No.** | **Questions** | **Definitely True**  **1** | **Mostly**  **True**  **2** | **Not**  **Sure**  **3** | **Mostly**  **False**  **4** | **Definitely**  **False**  **5** |
| --- | --- | --- | --- | --- | --- | --- |
| 33.1 | I am somewhat ill. |  |  |  |  |  |
| 33.2 | I am as healthy as anybody I know. |  |  |  |  |  |
| 33.3 | My health is excellent. |  |  |  |  |  |
| 33.4 | I have been feeling bad lately. |  |  |  |  |  |

34. How has the quality of your life been during the **past 4 weeks**? That is, how have things been going for you?

__1. Very well; could hardly be better

__2. Pretty good

__3. Good and bad parts about equal

__4. Pretty bad

__5. Very bad; could hardly be worse

35. How would you rate your physical health and emotional condition now compared to **4 weeks ago**?

__1. Much better

__2. A little better

__3. About the same

__4. A little worse

__5. Much worse

**Section 4: Diseases**

*This section asks about your diseases*

Have you ever had any of the following diseases? ( You can choose more than one answer)

| **No.** | **Diseases** | **Yes** | **No** |
| --- | --- | --- | --- |
| 36 | Hypertension |  |  |
| 37 | Chronic renal failure |  |  |
| 38 | Diabetes Mellitus |  |  |
| 39 | Hypercholesterolemia |  |  |
| 40 | Other Disease (Specify) ________________ |  |  |

***Thank you very much for taking time to answer the questionnaire.***
