# Supplementary material for: Socio-behavioral risk factors among older adults living with HIV in Thailand
Source: PLoS One. 2017 Nov 14;12(11):e0188088. doi: 10.1371/journal.pone.0188088 (PMC5685602; doi:10.1371/journal.pone.0188088)
Supplement: S3 File — (PDF) [file pone.0188088.s004.pdf]

Site ☐☐ - PID ☐☐☐

แบบสรุปข้อมูลทางคลีนิกจากเวชระเบียน
(สำหรับเจ้าหน้าที่โรงพยาบาลกรอก)

Date of Record: ☐☐ / ☐☐ /25☐☐ (วัน / เดือน / ปี พ.ศ.)

ชุด 1 หน้า 1

ข้อมูลอาสาสมัคร

1. เพศ : ☐ 1. ชาย ☐ 2. หญิง 2. วัน เดือน ปี เกิด : ☐☐ / ☐☐ / ☐☐☐☐ (วัน / เดือน / ปี พ.ศ.) 3. อายุ : ☐☐ ปี

Cardiovascular risk factor

I . Atherosclerotic risk factors	1.Yes	2.No
1. Hypertension (BP ≥ 140/90mmHg or currently treated with antihypertensive agents)	☐	☐
2. Chronic kidney disease stage I-V (Proteinuria ≥ 1+ or cGFR<60 ml/m)	☐	☐
3. Dyslipidemia or currently treated with lipid modifying agent	☐	☐
4. Diabetes Mellitus	☐	☐
5. Other disease : if Yes specify: ____________________	☐	☐

II. Previous history of cardiovascular events (year of events)

	1.Yes	2.No	If Yes, year of events
1. Chronic stable angina	☐	☐	☐☐☐☐
2. Myocardial infarction	☐	☐	☐☐☐☐
3. Unstable angina	☐	☐	☐☐☐☐
4. Stroke (ischemic)	☐	☐	☐☐☐☐
5. Transient ischemic attack	☐	☐	☐☐☐☐
6. Peripheral arterial disease	☐	☐	

If Q.6 = yes , continue q 6.1 - 6.4	1.Yes	2.No	If Yes, year of events	
6.1 intermittent claudication	☐	☐	☐☐☐☐	----> เดินได้ระยะ ______ เมตร ก่อนปวดขา
6.2 rest pain	☐	☐	☐☐☐☐	
6.3 gangrene	☐	☐	☐☐☐☐	
6.4 chronic ulcer (> 4 wks)	☐	☐	☐☐☐☐	

	1.Yes	2.No	If Yes, year of events
7. Chronic heart failure	☐	☐	☐☐☐☐
8. History of arrhythmia	☐	☐	☐☐☐☐

III. Previous history of cardiovascular intervention (year of intervention)

	1.Yes	2.No	If Yes, year of intervention
1.Percutaneous coronary intervention	☐	☐	☐☐☐☐
2.Coronary artery bypass graft(CABG)	☐	☐	☐☐☐☐
3.Aortic surgery	☐	☐	☐☐☐☐
4.Peripheral artery angioplasty	☐	☐	☐☐☐☐
5. Peripheral artery bypass surgery	☐	☐	☐☐☐☐
6. Amputation of ischemic limbs	☐	☐	☐☐☐☐
7. Carotid stenting	☐	☐	☐☐☐☐
8. Carotid endarterectomy	☐	☐	☐☐☐☐

Site ☐☐ - PID ☐☐☐

แบบสรุปข้อมูลทางคลีนิกจากเวชระเบียน (สำหรับเจ้าหน้าที่โรงพยาบาลกรอก)

Date of visit : ☐☐ (วัน) / ☐☐ (เดือน) /25 ☐☐ (ปี พ.ศ.)

ชุด 1 หน้า 2

Laboratory

ข้อมูลล่าสุดเฉพาะภายในระยะเวลา 3 เดือนที่ผ่านมา

	1.Yes	2.No	if yes, Value		วัน / เดือน / ปี พ.ศ.
1. Fasting blood sugar	☐	☐	☐☐☐☐ mg/dl	→	☐☐/☐☐/25☐☐
2. Random blood sugar	☐	☐	☐☐☐☐ mg/dl	→	☐☐/☐☐/25☐☐
3. HbA1C	☐	☐	☐☐☐.☐ %	→	☐☐/☐☐/25☐☐
4. Total Cholesterol	☐	☐	☐☐☐☐ mg/dl	→	☐☐/☐☐/25☐☐
5. Triglyceride	☐	☐	☐☐☐☐ mg/dl	→	☐☐/☐☐/25☐☐
6. HDL-C	☐	☐	☐☐☐☐ mg/dl	→	☐☐/☐☐/25☐☐
7. Directed LDL	☐	☐	☐☐☐☐ mg/dl	→	☐☐/☐☐/25☐☐
8. Serum Creatinine	☐	☐	☐☐☐.☐ mg/dl	→	☐☐/☐☐/25☐☐
9. Urine protein	☐	☐	(0-4, trace=0) ☐	→	☐☐/☐☐/25☐☐

Admission history

ในช่วง 12 เดือนที่ผ่านมา อาสาสมัครเคยนอนรักษาในโรงพยาบาลหรือไม่ ☐ 1. ใช่ ☐ 2. ไม่ใช่

1. **ถ้าใช่** กรุณาระบุการวินิจฉัยหลักที่ทำให้จำเป็นต้องนอนรักษาในโรงพยาบาลแต่ละครั้ง

ครั้งที่ 1 : ____________________

ครั้งที่ 2 : ____________________

ครั้งที่ 3 : ____________________

Site ☐☐ - PID ☐☐☐

แบบสรุปข้อมูลทางคลีนิกจากเวชระเบียน (สำหรับเจ้าหน้าที่โรงพยาบาลกรอก)

Date of visit : ☐☐ / ☐☐ /25 ☐☐ (วัน / เดือน / ปี พ.ศ.)

เฉพาะสำหรับอาสามัครกลุ่มที่ติดเชื้อเอชไอวี

1. HIV Positive date : ☐☐ / ☐☐ /25 ☐☐ (วัน / เดือน / ปี พ.ศ.)

2. History of OI : อาสาสมัครเคยป่วยเป็นโรคต่อไปนี้หรือไม่

2.1 Pneumonia : PCP	☐ 1.ใช่	☐ 2.ไม่ใช่
2.2 Toxoplasmosis	☐ 1.ใช่	☐ 2.ไม่ใช่
2.3 Tuber culosis	☐ 1.ใช่	☐ 2.ไม่ใช่
2.4 Oral candidiasis	☐ 1.ใช่	☐ 2.ไม่ใช่
2.5 Cryptococcosis	☐ 1.ใช่	☐ 2.ไม่ใช่

2.6 อื่นๆ ระบุ ____________________

Laboratory *(ครั้งล่าสุด)*

	1.Yes	2.No	if yes, Value	วัน / เดือน / ปี พ.ศ.
1. Absolute CD4+ lated	☐	☐	☐☐☐☐ cells/mm^3 →	☐☐ / ☐☐ /25 ☐☐
1.1 Percentage CD4+	☐	☐	☐☐.☐ %	
2. Plasma viral load	☐	☐	☐ less than ☐ equal to ☐ greater than<br>☐☐☐☐☐☐☐ copies/mL →	☐☐ / ☐☐ /25 ☐☐

ARV Treatment : อาสาสมัครเคยได้รับยาต้านไวรัสหรือไม่ ☐ 1. ใช่ ☐ 2.ไม่ (เสร็จสิ้นการกรอกข้อมูล)

1. **ถ้าใช่** เคยได้ยาต้านไวรัสมานานแค่ไหน ☐☐ ปี ☐☐ เดือน ☐☐ วัน

2. Antiretroviral Treatment Regimen *(Lastest drug regimen):*

Regimen Start Date : ☐☐ / ☐☐ / ☐☐☐☐ (วัน / เดือน / ปี พ.ศ.)

Item 1

ARV Code: ☐☐

Dose : ☐☐☐☐ mg.

(Record generic name:)

Name : ....

Frequency : ☐ 1-prn ☐ 2-qd ☐ 3-tid ☐ 4-qhs ☐ 5-qxh:every ☐☐ hrs ☐ 6-once ☐ 7-bid ☐ 8-qid ☐ 9-other,specify: ______________

Item 2

ARV Code: ☐☐

Dose : ☐☐☐☐ mg.

(Record generic name:)

Name : ....

Frequency : ☐ 1-prn ☐ 2-qd ☐ 3-tid ☐ 4-qhs ☐ 5-qxh:every ☐☐ hrs ☐ 6-once ☐ 7-bid ☐ 8-qid ☐ 9-other,specify: ______________

Item 3

ARV Code: ☐☐

Dose : ☐☐☐☐ mg.

(Record generic name:)

Name : ....

Frequency : ☐ 1-prn ☐ 2-qd ☐ 3-tid ☐ 4-qhs ☐ 5-qxh:every ☐☐ hrs ☐ 6-once ☐ 7-bid ☐ 8-qid ☐ 9-other,specify: ______________

ARV Code List

01 Abacavir (ABC, Ziagen)
02 Abacavir/Lamivudine (ABC/3TC, Epzicom)
03 Amprenavir (APV; Agenerase)
04 Atazanavir (ATV; Reyataz)
05 Combivir (Lamivudine/AZT; 3TC/ZDV)
06 Delavirdine mesylate (DLV; Rescriptor)
07 Didanosine (ddl; Videx)
08 Didanosine Delayed Release Capsules (ddl-EC; VidexEC)
09 Efavirenz (EFV; Sustiva; Stocrin)
10 Emtricitabine (FTC; Emtriva, Coviracil)
11 Emtricitabine/Tenofovir (FTC/TDF; Truvada)
12 Enfuvirtide, (ENF; Fuzeon)
13 Fosamprenavir (FPV, Lexiva)
14 Indinavir (IDV; Crixivan)
15 Lamivudine (3TC; Epivir)
16 Lamivudine/Zidovudine/Abacavir (3TC/AZT/ABC;Trizivir)
17 Lopinavir/ritonavir (LPV/RTV; Kaletra)
18 Nelfinavir (NFV; Viracept)
19 Nevirapine (NVP; Viramune)
20 Ritonavir (RTV; Norvir)
21 Saquinavir Hard-Gel Capsules (SQV; Invirase)
22 Saquinavir Soft-Gel Capsules (SQV; Fortovase)
23 Stavudine (d4T; Zerit)
24 Stavudine Extended Release Capsules (d4T XR; Zerit XR)
25 Tenofovir Disproxil Fumarate (TDF; Viread)
26 Zalcitabine (ddC; Hivid)
27 Zidovudine (AZT, ZDV, Retrovir)
28 Tipranavir (Aptivus)
29 GPOVIR (d4T/3TC/NVP)
30 Atripla (EFV/Emtricitabine/Tenofovir DF)
33 Lopinavir/ritonavir (Aluvia)
34 GPOVIR Z (AZT/3TC/NVP)
99 Other

Dose : Record the total milligram for one dose.

- A dose is the number of pills prescribed at an interval (e.g., two 150 mg pills bid equals two doses per day of 300 mg.)
- For combination drugs, such as Combivir, line through the dose boxes and date and initial.

Frequency : Below is a list of common frequency abbreviations. If the “other, specify: box is marked,”

**prn** - as needed	**qd** - every day	**tid** - three times daily	**qhs** - at bedtime
**once** - one time	**bid** - two times daily	**qid** - four times daily	**qxh** - every x hours

PTID ☐☐☐☐ Initial ☐-☐

แบบบันทึกผลการตรวจร่างกาย
(สำหรับเจ้าหน้าที่โครงการวิจัย)

Date of visit : ☐☐ / ☐☐ /25 ☐☐
วัน เดือน ปี พ.ศ.

ชุด 2 หน้า 1

Physical Examination (จากเครื่องวัด ABI อัตโนมัติ)

1. Body weight : ☐☐☐.☐ kg.
2. Height ☐☐☐.☐ cm.
3. Waist circumference ☐☐☐.☐ cm.

Investigation

1. Systolic blood pressure ☐☐☐ mmHg
2. Diastolic blood pressure ☐☐☐ mmHg
3. Heart rate ☐☐☐ bpm
4. Ankle-brachial index *Left* ☐.☐☐ *Right* ☐.☐☐
5. CAVI *Left* ☐☐.☐☐ *Right* ☐☐.☐☐
